# Adipokines as Possible New Predictors of Cardiovascular Diseases: A Case Control Study

**DOI:** 10.1155/2012/253428

**Published:** 2011-08-22

**Authors:** Laura Pala, Matteo Monami, Silvia Ciani, Ilaria Dicembrini, Alessandro Pasqua, Anna Pezzatini, Paolo Francesconi, Barbara Cresci, Edoardo Mannucci, Carlo Maria Rotella

**Affiliations:** ^1^SOD of Endocrinology, AOUC, 50139 Florence, Italy; ^2^Unit of Gerontology and Geriatrics, Department of Critical Care Medicine and Surgery, University of Florence, 50139 Florence, Italy; ^3^Section of Endocrinology, Department of Clinical Pathophysiology, University of Florence, Viale P ieraccini 6, 50139 Florence, Italy; ^4^Epidemiology Unit, Local Health Unit 10, Florence, Italy; ^5^Diabetic Agency, AOUC, 50139 Florence, Italy

## Abstract

*Background and Aims*. The secretion of several adipocytokines, such as adiponectin, retinol-binding protein 4 (RBP4), adipocyte fatty acid binding protein (aFABP), and visfatin, is altered in subjects with abdominal adiposity; these endocrine alterations could contribute to increased cardiovascular risk. The aim of the study was to assess the relationship among adiponectin, RBP4, aFABP, and visfatin, and incident cardiovascular disease. *Methods and Results*. A case-control study, nested within a prospective cohort, on 2945 subjects enrolled for a diabetes screening program was performed. We studied 18 patients with incident fatal or nonfatal IHD (Ischemic Heart Disease) or CVD (Cerebrovascular Disease), compared with 18 matched control subjects. Circulating adiponectin levels were significantly lower in cases of IHD with respect to controls. Circulating RBP4 levels were significantly increased in CVD and decreased in IHD with respect to controls. Circulating aFABP4 levels were significantly increased in CVD, while no difference was associated with IHD. Circulating visfatin levels were significantly lower in cases of both CVD and IHD with respect to controls, while no difference was associated with CVD. *Conclusions*. The present study confirms that low adiponectin is associated with increased incidents of IHD, but not CVD, and suggests, for the first time, a major effect of visfatin, aFABP, and RBP4 in the development of cardiovascular disease.

## 1. Introduction

Overweight and obesity are associated with a different secretion rate of several adipocytokines, such as reduced adiponectin [1] and increased retinol-binding protein 4 (RBP4) [2], adipocyte fatty acid binding protein (aFABP) [3], and visfatin [4]. aFABP plays an important role in maintaining glucose and lipid homeostasis. aFABP has been primarily regarded as an adipocyte- and macrophage-specific protein, but recent studies suggest that it may be more widely expressed [5]. Such endocrine modifications could be responsible, at least partly, for the increased cardiovascular risk associated with excess fat mass [6]. In particular, low adiponectin levels have been reported to be associated with increased incidence of myocardial infarction in men [7], although other groups did not find such association in women [8]. In elderly, RBP4 concentrations were associated with Metabolic Syndrome (MetS) and its components in both genders, and prior cerebrovascular disease in men [9].

A recent study was undertaken to determine plasma RBP4 and adiponectin levels in subjects with cerebral infarction and showed that adiponectin and hypertension were independent factors contributing to cerebral infarction; moreover, it has been shown that plasma RBP4 levels in the subjects with cerebral infarction were significantly greater than those in control subjects [10]. In addition, it was reported that visfatin is capable of reducing myocardial injury when administered at the time of myocardial reperfusion in both in situ murine heart and in the isolated murine cardiomyocytes [11].

The relationships between RBP4, aFABP, and visfatin, with respect to incident cardiovascular disease, have not been assessed, so far, in human models.

## 2. Patients and Methods

A case-control study was performed within the cohort enrolled in the Firenze-Bagno a Ripoli (FIBAR) study [12]. Briefly, all subjects aged 40–75 years without known diabetes were invited to participate to a diabetes screening program through newspaper and TV advertising, public conferences, and letters from family doctors. The local ethical committee approved the study, and each participant provided informed written consent. Venous blood samples for lipid profile and plasma glucose were collected in the morning, after overnight fast (>8 hrs). All subjects (*n* = 2945) underwent a standard oral glucose tolerance test (75 g in 50% water solution, with measurement of plasma glucose after 120 minutes). Blood pressure was measured in sitting position, after a 5-min rest using a mercury sphygmomanometer with a cuff of appropriate size; the mean of three measurements of systolic and diastolic blood pressure was considered for analysis. Patients were considered hypertensive if they were taking antihypertensive medication and/or if their office blood pressure was ≥140/90 mmHg [13]. Laboratory determinations were performed in the Central Laboratory of Careggi Hospital in Florence. Plasma glucose was measured by a glucose oxidase method; total and HDL cholesterol, and triglycerides were determined by an automated enzymatic method (Beckman, Brea, USA). Metabolic Syndrome was diagnosed according to NCEP criteria [14, 15]. The mean followup was 33.6 ± 6.7 months. Nonfatal cases requiring hospitalization and fatal of IHD (Ischemic Heart Disease) and CV (Cerebrovascular disease) were considered. Nonfatal cases were identified through the regional hospital discharge system using International Classification of Diseases (ICD) codes 410–414, 420–429, 798–799, and 430–434, 436–438 for IHD and CV, respectively. Fatal IHD and CV were identified through queries to the registry office of the municipalities of Florence and Bagno a Ripoli, selecting cases with the same ICD codes listed above. Incident cases of IHD (*n* = 9) and CV (*n* = 9) in individuals without any previous history of cardiovascular disease were compared with control subjects free of events from the same cohort. For each case, the first available subject matched for age (±2 years), gender, BMI (±2 Kg/m^2^), waist (±3 cm), and degree of glucose tolerance was selected as control. In cases and control subjects, serum adipocytokynes were measured using ELISA test for adiponectin (Linco Research, USA) and human Adipocyte aFABP (BioVendor GmbH, Germany), and EIA test for Visfatin and RBP4 (Phoenix Pharmaceuticals, USA). Statistical analysis was performed with SPSS 12.0.1. Data were expressed as mean ± SD, when normally distributed, and as median (quartiles), when their distribution was not normal. For comparisons between groups, unpaired two-tailed Student's *t*-tests and Mann-Whitney *U* tests were applied to normally and nonnormally distributed parameters, respectively. Stepwise logistic regression was used for multivariate analysis.

## 3. Results

**Figure 1:**
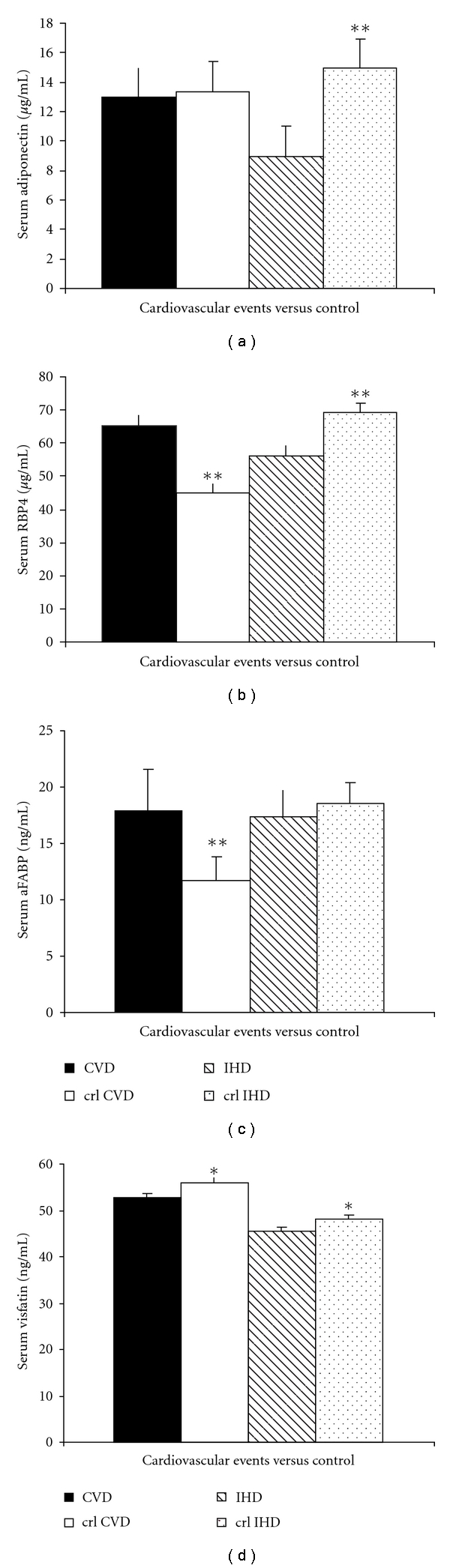
Comparison of mean adipocytokines levels between cases (black bars) of IHD and CVD and their controls (white bars). *P < 0.05, **P < 0.01. (a) Adiponectin is significantly reduced only in IHD with respect to controls. (b) RBP4 is reduced in a significant manner in IHD and increased significantly in CVD with respect to controls. (c) aFABP is significantly increased only in CVD with respect to controls. (d) Visfatin is significantly reduced both in IHD and CVD with respect to controls.

Cardiovascular events were detected in 18 subjects (9 IHD and 9 CV). The characteristics of cases of IHD and CVD as well as of respective controls are summarized in Table 1. Patients with IHD showed a significantly higher prevalence of hypertension, in comparison with their controls. No other significant differences between cases and controls were observed. Circulating adiponectin levels were significantly lower in cases of IHD with respect to controls (*P* = 0.021), while no difference was associated with CVD cases Figure 1(a)). The difference of adiponectin levels between cases of IHD and their controls retained statistical significance at multivariate analysis after adjustment for components of the metabolic syndrome, with an increased risk of IHD of 61% (2–161) (*P* < 0.05) for each decrement of 1 *μ*g/mL. Circulating RBP4 levels were significantly increased in cases of CVD with respect to controls (*P* = 0.001), while they resulted decreased in a significant manner in cases of IHD respect to controls (*P* = 0.006) (Figure 1(b)). Circulating aFABP levels were significantly increased in cases of CVD with respect to controls (*P* = 0.041), while no difference was associated with IHD (Figure 1(c)). Circulating visfatin levels were significantly lower in cases of both CVD and IHD with respect to controls (*P* = 0.014 and *P* = 0.035, resp.) (Figure 1(d)). 

## 4. Discussion

A number of different hormones produced by fat tissue have been identified in the last few years, and some of those molecules have been found to be associated with the regulation of insulin sensitivity. Since visceral adiposity and insulin resistance are known to be associated with increased cardiovascular risk [16], it could be speculated that some adipocytokines mediate this relationship. 

Our data have revealed different results among the different adipocytokines, accordingly to the observation that in cardiovascular events, predictive factors have a different weight: hypertension is more predictive for CVD, while hypercholesterolemia is more predictive for IHD. 

Moreover present data confirm that low adiponectin is an independent predictor of IHD, even after adjustment for components of the metabolic syndrome; on the other hand, adiponectin levels are not associated with the incidence of CVD. This confirms previous results [17, 18], highlighting pathophysiological differences between coronary and cerebrovascular disease.

RBP4 is secreted by adipose tissues and hepatocytes [19], and there are controversial reports regarding the effect of RBP4 on insulin resistance. It has been described that plasma RBP4 is increased in subjects with obesity, impaired glucose tolerance, and diabetes mellitus [20–24], but other studies did not support the relation between RBP4 and insulin resistance [25–30]. Recently, Sasaki et al. have shown increased levels of RBP4 in a sample of 58 Japanese with cerebral infarction which appeared to be significantly higher than in age- and sex-matched control subjects. Since present study is a case-control study with strong criteria of matching, the statistical relevance of such a small sample of patients is amplified by the method of the clinical analysis. Our data confirm this correlation between the RBP4 and CVD and for the first time demonstrate a correlation with IHD. 

Moreover, our study, for the first time, demonstrates that visfatin and aFABP have major effect on the development of cardiovascular disease, in particular, visfatin levels are significantly decreased both in cases of CVD and IHD, while increased aFABP levels are correlated with CVD but not with cases of IHD. On the basis of our observation, the measurement of adiponectin, RBP4, aFABP, and visfatin could be considered along with other cardiovascular risk factors in a larger clinical setting, for predicting the risk of developing major cardiovascular events.

##  Conflict of Interests

The authors declared that no conflict of interests exists.

## Figures and Tables

**Table 1:** Principal characteristics of the sample enrolled are described in the table.

	Ischemic heart diseases		Cerebrovascular disease	
	Controls	Cases	Controls	Cases
Number (women %)	9 (22.2%)	9 (22.2%)	9 (22.2%)	9 (22.2%)
Age (years)	62.9 ± 4.2	63.6 ± 4.9	65.5 ± 11.8	65.0 ± 11.7
Waist (cm)	98.8 ± 6.8	98.6 ± 6.7	94.9 ± 9.3	93.9 ± 9.0
BMI (kg/m^2^)	28.0 ± 1.7	28.2 ± 2.5	25.1 ± 2.4	24.7 ± 3.0
Total colesterol (mmol/L)	5.39 ± 0.83	6.21 ± 0.9	5.38 ± 0.82	5.8 ± 1.55
HDL colesterol (mmol/L)	1.26 ± 0.38	1.41 ± 0.5	1.65 ± 0.67	1.45 ± 0.43
Triglyceride (mmol/L)	1.19 (1; 2.67)	1.8 (1.42; 3.42)	1.7 (1.18; 2.88)	1.17 (0.96; 1.64)
Fasting glycemia (mmol/L)	5.52 ± 0.89	5.99 ± 1.64	4.98 ± 0.8	5.77 ± 0.91
Diabetes mellitus (%)	22.2	22.2	11.1	11.1
Hypertension* (%)	33.3	77.8	66.7	66.7
High fasting glycemia* (%)	11.1	22.2	11.1	11.1
High waist* (%)	55.6	68.7	66.7	44.4
Low HDL colesterol*(%)	33.3	22.2	11.1	11.1
Hypertriglyceridaemia* (%)	44.4	55.6	22.2	33.3

*as defined by NCEP criteria.

## References

[1] Arita Y, Kihara S, Ouchi N (1999). Paradoxical decrease of an adipose-specific protein, adiponectin, in obesity. *Biochemical and Biophysical Research Communications*.

[2] Yang Q, Graham TE, Mody N (2005). Serum retinol binding protein 4 contributes to insulin resistance in obesity and type 2 diabetes. *Nature*.

[3] Damcott CM, Moffett SP, Feingold E (2004). Genetic variation in fatty acid-binding protein-4 and peroxisome proliferator-activated receptor *γ* interactively influence insulin sensitivity and body composition in males. *Metabolism*.

[4] Pagano C, Pilon C, Olivieri M (2006). Reduced plasma visfatin/pre-B cell colony-enhancing factor in obesity is not related to insulin resistance in humans. *Journal of Clinical Endocrinology and Metabolism*.

[5] Elmasri H, Karaaslan C, Teper Y (2009). Fatty acid binding protein 4 is a target of VEGF and a regulator of cell proliferation in endothelial cells. *FASEB Journal*.

[6] Nakamura Y, Shimada K, Fukuda D (2004). Implications of plasma concentrations of adiponectin in patients with coronary artery disease. *Heart*.

[7] Pischon T, Girman CJ, Hotamisligil GS, Rifai N, Hu FB, Rimm EB (2004). Plasma adiponectin levels and risk of myocardial infarction in men. *Journal of the American Medical Association*.

[8] Matsubara M, Maruoka S, Katayose S (2002). Decreased plasma adiponectin concentrations in women with dyslipidemia. *Journal of Clinical Endocrinology and Metabolism*.

[9] Ingelsson E, Sundström J, Melhus H (2009). Circulating retinol-binding protein 4, cardiovascular risk factors and prevalent cardiovascular disease in elderly. *Atherosclerosis*.

[10] Sasaki M, Otani T, Kawakami M, Ishikawa S (2010). Elevation of plasma retinol-binding protein 4 and reduction of plasma adiponectin in subjects with cerebral infarction. *Metabolism*.

[11] Lim SY, Davidson SM, Paramanathan AJ, Smith CCT, Yellon DM, Hausenloy DJ (2008). The novel adipocytokine visfatin exerts direct cardioprotective effects. *Journal of Cellular and Molecular Medicine*.

[12] Mannucci E, Ognibene A, Sposato I (2003). Fasting plasma glucose and glycated haemoglobin in the screening of diabetes and impaired glucose tolerance. *Acta Diabetologica*.

[13] Chalmers J, MacMahon S, Mancia G (1999). 1999 World Health Organization-International Society of Hypertension Guidelines for the management of hypertension. Guidelines sub-committee of the World Health Organization. *Clinical and Experimental Hypertension*.

[14] Alberti KGMM, Zimmet P, Shaw J (2006). Metabolic syndrome—a new world-wide definition. A consensus statement from the International Diabetes Federation. *Diabetic Medicine*.

[15] Cleeman JI (2001). Executive summary of the third report of the National Cholesterol Education Program (NCEP) expert panel on detection, evaluation, and treatment of high blood cholesterol in adults (adult treatment panel III). *Journal of the American Medical Association*.

[16] Yusuf S, Hawken S, Ôunpuu S (2005). Obesity and the risk of myocardial infarction in 27,000 participants from 52 countries: a case-control study. *The Lancet*.

[17] Chen MP, Tsai JCR, Chung FM (2005). Hypoadiponectinemia is associated with ischemic cerebrovascular disease. *Arteriosclerosis, Thrombosis, and Vascular Biology*.

[18] Kojima S, Funahashi T, Sakamoto T (2003). The variation of plasma concentrations of a novel, adipocyte derived protein, adiponectin, in patients with acute myocardial infarction. *Heart*.

[19] Yang Q, Graham TE, Mody N (2005). Serum retinol binding protein 4 contributes to insulin resistance in obesity and type 2 diabetes. *Nature*.

[20] Graham TE, Yang Q, Blüher M (2006). Retinol-binding protein 4 and insulin resistance in lean, obese, and diabetic subjects. *New England Journal of Medicine*.

[21] Takebayashi K, Suetsugu M, Wakabayashi S, Aso Y, Inukai T (2007). Retinol binding protein-4 levels and clinical features of type 2 diabetes patients. *Journal of Clinical Endocrinology and Metabolism*.

[22] Cho YM, Youn BS, Lee H (2006). Plasma retinol-binding protein-4 concentrations are elevated in human subjects with impaired glucose tolerance and type 2 diabetes. *Diabetes Care*.

[23] Qi Q, Yu Z, Ye X (2007). Elevated retinol-binding protein 4 levels are associated with metabolic syndrome in Chinese people. *Journal of Clinical Endocrinology and Metabolism*.

[24] Janke J, Engeli S, Boschmann M (2006). Retinol-binding protein 4 in human obesity. *Diabetes*.

[25] Stefan N, Hennige AM, Staiger H, Schleicher E, Fritsche A, Häring HU (2007). Circulating retinol-binding protein-4, insulin sensitivity, insulin secretion, and insulin disposition index in obese and nonobese subjects: response to Broch et al.. *Diabetes Care*.

[26] Promintzer M, Krebs M, Todoric J (2007). Insulin resistance is unrelated to circulating retinol binding protein and protein C inhibitor. *Journal of Clinical Endocrinology and Metabolism*.

[27] Lewis JG, Shand BI, Elder PA, Scott RS (2008). Plasma retinol-binding protein is unlikely to be a useful marker of insulin resistance. *Diabetes Research and Clinical Practice*.

[28] Yao-Borengasser A, Varma V, Bodles AM (2007). Retinol binding protein 4 expression in humans: relationship to insulin resistance, inflammation, and response to pioglitazone. *Journal of Clinical Endocrinology and Metabolism*.

[29] Bajzová M, Kováciková M, Vítková M (2009). Retinol-binding protein 4 expression in visceral and subcutaneous fat in human obesity. *Physiological Research*.

[30] von Eynatten M, Lepper PM, Liu D (2007). Retinol-binding protein 4 is associated with components of the metabolic syndrome, but not with insulin resistance, in men with type 2 diabetes or coronary artery disease. *Diabetologia*.

